# Rapid detection of the common avian leukosis virus subgroups by real-time loop-mediated isothermal amplification

**DOI:** 10.1186/s12985-015-0430-1

**Published:** 2015-11-24

**Authors:** Hao Peng, Lili Qin, Yuyu Bi, Peikun Wang, Guangzhen Zou, Jun Li, Yongli Yang, Xingfu Zhong, Ping Wei

**Affiliations:** Institute for Poultry Science and Health, Guangxi University, Nanning, 530004 China; Guangxi Key Laboratory of Animal Vaccines and Diagnostics, Guangxi Veterinary Research Institute, 51 Youai Bei Road, Nanning, 530001 China

**Keywords:** ALV, LAMP, Rapid detection

## Abstract

**Background:**

Subgroups A, B, E and J are the major subgroups of avian leukosis virus (ALV) infecting chickens. ALV infection has become endemic in China and has a significant negative effect on the poultry industry. Consequently, there is an urgent need for a specific, sensitive and rapid method for diagnosis and eradication of ALV. Therefore, we developed a simple and rapid real-time loop-mediated isothermal amplification (LAMP) reaction for the timely detection of the common ALV subgroups, whereby the amplification can be obtained in 35 min under isothermal conditions at 63 °C, ability to specific, sensitive and rapid detect all the common ALV subgroups.

**Methods:**

A set of four specific primers was designed to target the sequences of the *pol* gene of ALV, and the loop-mediated isothermal amplification (LAMP) assay were developed and compared with PCR and virus isolation methods.

**Results:**

The results from specificity of the LAMP assay showed that only target ALVs DNA was amplified. The LAMP assay demonstrated a sensitivity of 20 copies/reaction of ALV DNA, which was 10 times higher than the conventional PCR measurement. To further evaluate the reliability of the method, the assay was evaluated with ALV DNA from a panel of 81 clinical samples suspected of ALV infection. The results verify that the LAMP method was more sensitive than the conventional PCR and virus isolation method.

**Conclusion:**

In conclusion, the developed LAMP assay was a simple, inexpensive, sensitive method for the rapid detection of the most common subgroups of ALV, and it provided a useful and practical tool in the eradication program for ALV in the poultry industry.

## Background

Avian leukosis virus (ALV) is an economically important poultry pathogen and its infection may result in low productivity and tumor mortality in chickens. According to the viral envelope glycoprotein antigenic structure, host range and mutual interference between different strains cultured in cells, ALV can be classified as ten subgroups, A-J. Subgroups A-E and J exist in chickens. ALV- A, B and J are the most common exogenous subgroups that cause chicken tumors, but subgroups C, D appear to be rare [1]. Subgroup E is an endogenous leukosis virus, which has low or no pathogenicity to chickens directly, but studies have shown that chickens infected with ALV-E remained viremic with exogenous virus longer and developed neoplasm at a much higher frequency than did the control chickens not infected with ALV-E [2]. For best results, all the common ALV subgroups needed to be eradicated from the chicken breeder flocks. Currently, to determine these pathogens in large quantities, a preliminary test is done to see whether ALVs exist and then, if necessary, primers specified for every subgroup are used to detect the positive samples.

Since the Nationwide Eradication Program (NEP) for ALV in chicken breeder farms had not been instituted in China until 2008, ALV infection in chickens had caused serious problems. Over the past decade, many myeloid tumor cases induced by ALV have been reported, especially involving ALV-J [3]. At first, it was only found in white meat-type breeders. Later, it was discovered that there were a growing number of AL cases in layers and local meat-type chickens. Over the past major economic losses were reported [4]. In recent years, the reports of leukemia/ hemangioma in post-laying chickens have increased significantly all over the country. Virus isolation and identification showed that leukemia/ hemangioma is mainly caused by ALV-J, but chickens are coinfected with the ALV-A or/and ALV-B at the same time [5]. In conclusion, ALV-A, ALV-B, and ALV-J were identified as the most common ALVs pathogens in the poultry industry and ALV-E co-infection was found to increase the pathogenicity of exogenous infection. An eradication program for ALVs has been developed and is being used on quite a few chicken farms in China since the NEP started in 2008. Unlike in the United States, where there are relatively few major breeding companies, in China there are more smaller poultry breeding companies and many of these produce “yellow chickens” of local breeds. These companies are in great need of an eradication program for ALV, but, in fact, they lack the money, technology and professional staff to obtain one. It appears that only a simple, rapid, and inexpensive detection assay would be suitable for these companies.

Some methods, such as PCR assays, were developed for the detection of ALV [6]. However, while these methods for specific, sensitive, and rapid diagnosis appear to be promising, PCR requires skilled personnel and specialized high-cost instruments to observe the test results [7]. Virus isolation is the gold standard method for the detection of ALV, but it is very difficult to see this as practical in most of the smaller poultry breeding companies because it would require skilled personnel and specialized high-cost instruments. Also, it takes about 7–9 days after CEFs or DF-1 cell cultures are infected by the virus before they can be detected. In addition the immunofluorescence assay (IFA) based on the cell cultures takes further time. An enzyme-linked immunosorbent assay (ELISA) kit targeting the viral group-specific antigen (*p*27) was used in some large poultry breeder farms of China by those wanting to establish exogenous ALV-free breeder flocks when the NEP started. But, this assay is expensive for smaller companies and is time-consuming, especially if there is no costly ELISA plate-washing machine. Also, it results in a considerable proportion of false-positive results because some endogenous retroviruses like EAV-HP family may express *p*27. Therefore, the ELISA assay is not practical in all cases [8]. As a result, there is high demand for simple, simple and rapid molecular tests to supplement existing methods.

Loop-mediated isothermal amplification (LAMP) was developed by Notomi et al [9], based upon nucleic acid specificity and rapid amplification and has developed into a competitive molecular biology tool due to its specificity, simplicity, speed, and economical efficiency. The LAMP reaction is carried out at a constant temperature without the DNA template denaturation, annealing, and extension PCR cycles in a specific instrument [10]. In addition, the results can be easily identified through the naked eye [11]. The LAMP assay has been widely applied in clinical diagnosis of epidemic viruses [12–14], bacteria [15–17] and parasites [18], as well as in sex determination of embryos [19] and other applications. Although there was already a LAMP assay for rapid detection of ALV-J reported [20], the NEP for ALV in China needed to detect all the common subgroups of exogenous ALVs including the most common subgroups A, B, E and J and to eradicate them as possible in the production of poultry. Compared with the existing methods, the most efficient process may be a preliminary test showing whether any ALVs exist, and then if necessary, the use of primers specified for every subtype to detect the positive samples further. Thus, we have developed a LAMP assay for the simple, sensitive, and rapid detection of all the common ALV subgroups found in chickens, with the goal of helping to improve the NEP. First, we designed four sets of primers for common ALVs detection and optimized the reaction conditions of LAMP. Secondly, the specificity and sensitivity of the LAMP assay were evaluated. Finally, the reliability of the LAMP assay was evaluated on the detection of 81 clinical samples and compared to the conventional PCR method and virus isolation method. To ensure the accuracy of the results the assay was real-time monitored by the Loopamp real-time turbidimeter (LA320-C, Eiken Chemical Co., Ltd., Tokyo, Japan) at 400 nm [21].

## Results

### Specificity of the LAMP assay

**Fig. 1:**
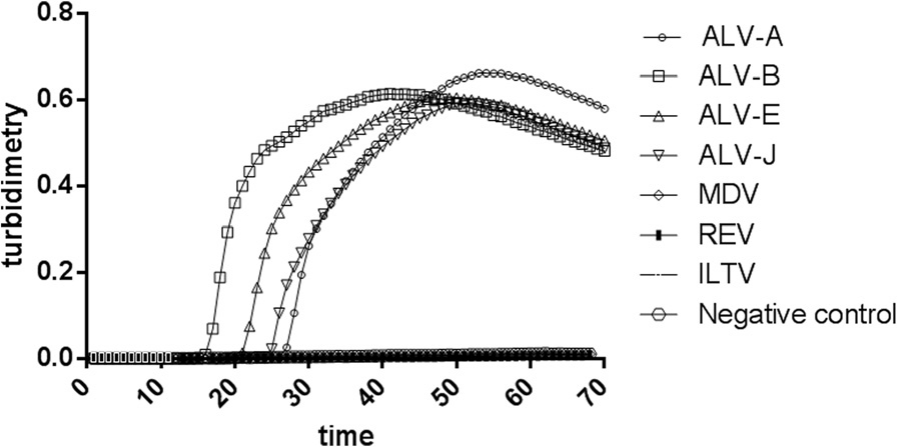
Specificity of the LAMP measure for the detection of common exogenous ALV. Specificities of the LAMP assay were monitored by real-time measurement of turbidity (LA-320c. Positive reactions were defined as those samples having a threshold value of greater than 0.2 within 60 min. Positive reactions were only observed in the ALV-A virus (round), the ALV-B virus (square), the ALV-E virus(regular triangle) and the ALV-J virus (inverted triangle)

**Fig. 2:**
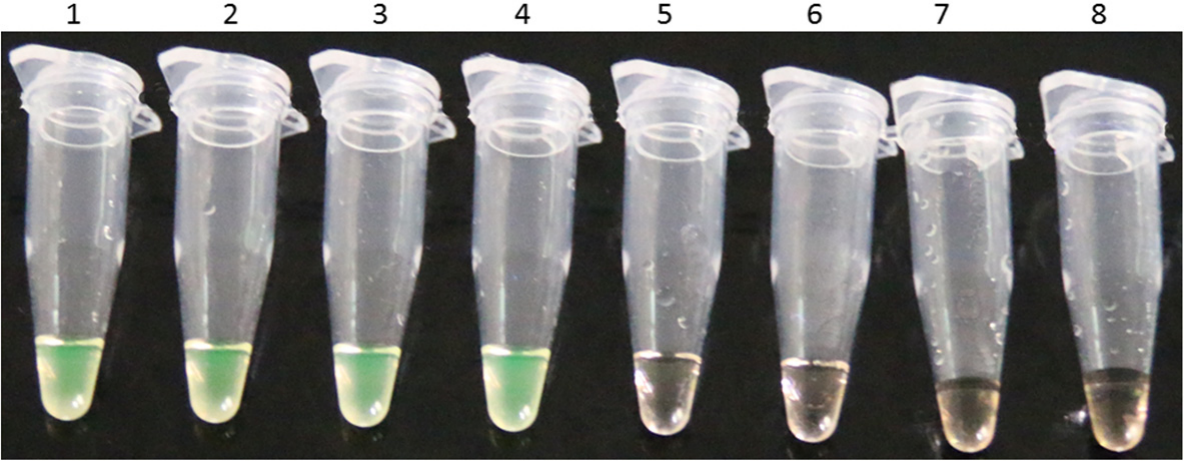
Visual detection of LAMP assay specificity. The tubes represent ALV strains and the negative controls used in the visual inspection of LAMP assay specificity test. 1, ALV-A; 2, ALV-B; 3, ALV-E; 4, ALV-J; 5, MDV; 6, REV; 7, ILTV; 8, Negative control

The specificity of the LAMP assay was determined with the specific samples of ALV subgroups A, B, E and J, Marek’s disease virus (MDV), reticuloendotheliosis virus (REV) and avian infectious laryngotrachetis virus (ILTV). All samples were purified and contained no other virus DNA as confirmed by classiscal PCR methods. ALV subgroups A, B, E , J samples gave a positive LAMP reaction in about 25 min, while no rising curve of turbidity was seen with the samples of MDV, REV or ILTV. The data are shown in Fig. 1. Besides observations by real-time turbidimetry, we also used visual inspection to determine a positive reaction in a temperature-controlled water bath. After addition of commercial calcein dye to the terminated reaction at about 35 min when the LAMP reaction terminated according to the optimized conditions by real-time turbidimetry, the results were obtained with UV light trans-illumination. The results of visual detection of the LAMP assay specificity showed that ALV subgroups A, B, E , J samples yielded a positive color change to a yellowish-green color, whereas the samples of MDV, REV or ILTV seem clear under UV light indicating a negative reaction (Fig. 2).

### LAMP assay sensitivity

**Fig. 3:**
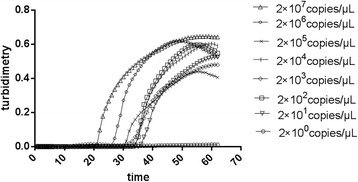
Sensitivity of the LAMP detections. Turbidity was monitored by the Loopamp real-time turbidimeter at 400 nm. The detection limits for normal exogenous ALV was 2 × 10^1^ copies/μl of positive samples

**Fig. 4:**
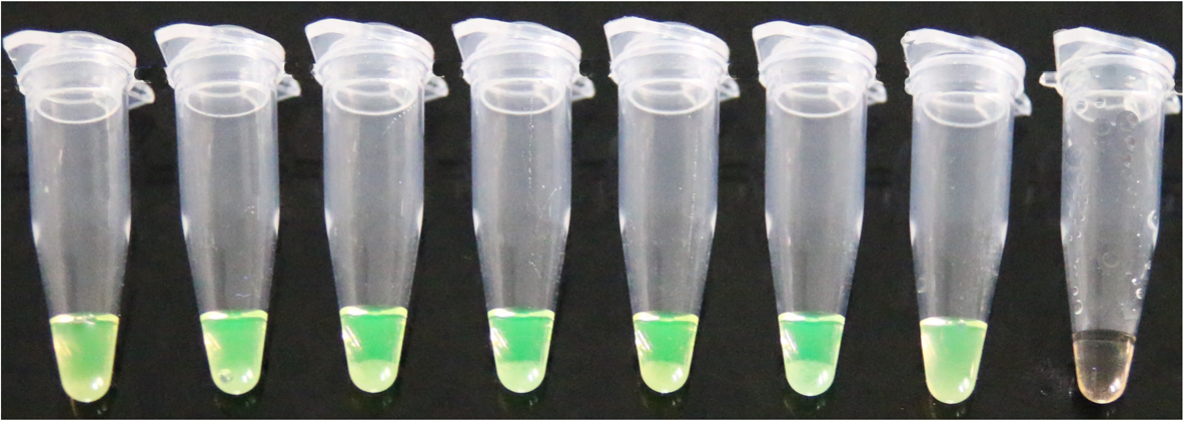
Visual detection of LAMP assay sensitivity. The tubes represent samples used in the visual inspection of LAMP assay sensitivity test. 1, 2 × 10^7^ copies/μl; 2, 2 × 10^6^ copies/μl; 3, 2 × 10^5^ copies/μl; 4, 2 × 10^4^ copies/μl; 5, 2 × 10^3^ copies/μl; 6, 2 × 10^2^ copies/μl; 7, 2 × 10^1^ copies/μl; 8, 2 × 10^0^ copies/μl

**Fig. 5:**
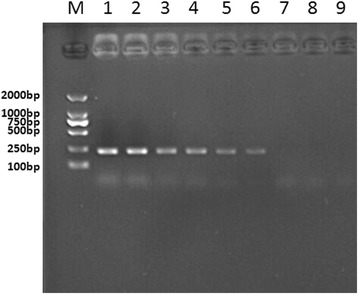
The PCR productions. The PCR products revealing a 226-bp specific amplicon for ALV were analyzed by 1.5 % agarose gel electrophoresis and stained with ethidium bromide. Lane M, DNA marker DL-2000 (CWBIO, Beijing); 1, 2 × 10^7^ copies/μl; 2, 2 × 10^6^ copies/μl; 3, 2 × 10^5^ copies/μl; 4, 2 × 10^4^ copies/μl; 5, 2 × 10^3^ copies/μl; 6, 2 × 10^2^ copies/μl; 7, 2 × 10^1^ copies/μl; 8, 2 × 10^0^ copies/μl; 9, Negative control

The recombinant plasmid was obtained by cloning a 226-bp fragment of ALV-J that was amplified with the primers ALV-F3 and ALV-B3 into the *p*MD-18 T vector (TaKaRa, Dalian, China). The concentration of the plasmid was judged by a UV spectrophotometer (Beckman UV-800, Beckman Coulter, USA). According to Lamien’s method [22] , the copy number was calculated as follows: number of copies = [amount (ng) × 6.022 × 10^23^]/[length(plasmid + insert) × 1 × 10^9^ × 650]. The plasmid containing the ALV-J *pol* gene fragment was serially diluted from 2 × 10^0^ to 2 × 10^7^ copies/μl. Then the sensitivity of the LAMP assay was compared with the conventional PCR method. The plasmids concentration was subjected to the LAMP assay and routine PCR respectively. The results showed that the detection limit of the LAMP assay monitored by real-time turbidimetry was about 20 copies (Fig. 3). The detection limit of the LAMP assay by direct visual detection was also 20 copies (Fig. 4). Therefore, we concluded that these two LAMP detection methods had the same sensitivity. For comparison purposes, PCR using ALV-F3 and ALV-B3 primers was also carried out. We observed that the limit of the PCR was 200 copies (Fig. 5). Then we repeated sensitivity test 2 times. There were no apparent differences in the least detectable amount except slight differences which could be neglected in the detection time. Consequently, the sensitivity of this LAMP assay was at least 10 times higher than that of the routine PCR method.

### Detection of ALV from clinical samples

A total of 81 tissue samples of suspected ALV infection were treated, extracted of DNA and detected by the LAMP method as described above. The positivity rate was 77.8 % (63/81) (Table 1). The same samples were subjected to conventional PCR and virus isolation, by the *p*27 detection on the supernatants of the cell cultures, and the positivity rates were 65.4 % (53/81) and 46.1 % (37/81), respectively (Table 1). Out of a total of 63 positive samples by the LAMP assay, 34 samples were also shown to be positive by both PCR and virus isolation. Another 29 samples were negative by either one or both methods. We tested LAMP and ELISA against PCR as the current reference method, and calculated their sensitivity (SEN), specificity (SPC), positive predictive value (PPV) and negative predictive value (NPV). Results are listed in Table 2. The results suggested that the LAMP assay was the most sensitive among the three methods and was able to effectively avoided false negative.

**Table 1:** Analysis of clinical samples

Location of the samples	Viral isolation (p27 detection)		PCR		LAMP	
	Positive samples/total samples	Positive rate	Positive samples/total samples	Positive rate	Positive samples/total samples	Positive rate
Nanning	14/20	70.0 %	11/20	55.0 %	15/20	75.0 %
Yulin	19/33	57.6 %	29/33	87.9 %	30/33	90.9 %
Qinzhou	1/7	14.3 %	2/7	28.6 %	4/7	57.1 %
Baise	1/9	11.1 %	3/9	33.3 %	5/9	55.6 %
Liuzhou	2/12	16.7 %	8/12	66.7 %	9/12	75.0 %
Total	37/81	46.1 %	53/81	65.4 %	63/81	77.8 %

**Table 2:** Sensitivity, specificity, positive predictive value and negative predictive value for the comparison between p27 assay and LAMP assay

	Sensitivity	Specificity	Positive predictive value	Negative predictive value
p27 assay	76.8 %	90.3 %	94.6 %	63.6 %
LAMP assay	100 %	73.7 %	84.1 %	100 %

## Discussion

ALV infection cases have been reported extensively for commercial layers and breeders over the past decade in China [23–26]. ALV- A, B, E and J are the most common pathogenic subgroups. An effective medication or vaccine against these ALVs is not currently available. As a result, the control or preventive procedures of ALV infections mainly depends upon early detection and limitation of the virus carriers to prevent the vertical and horizontal transmission. Virus isolation is regarded as the golden standard method for ALV diagnosis. However, it requires skilled personnel knowing complex cell culture procedures and obtaining the results takes more than 1 week [27]. It is too difficult to apply for the numerous smaller or mid-sized poultry breeding companies in China as opposed to the fewer, larger firms in the USA and many European countries. The *p*27 antigen ELISA assay is routinely used for the ALV diagnosis, but it also is expensive and time-consuming [28]. These are the reasons we have developed a specific, sensitive, inexpensive and real-time monitored LAMP assay for the detection of major ALV subgroups infected in chickens. This is the first report on the LAMP method being used for the rapid detection of the common ALVs.

The LAMP assay for ALV diagnosis was able to detect these ALV subgroups within about 30 min as a result of evaluation and optimization. By evaluating the sensitivity of the LAMP assay, we observed that the LAMP assay was 10-fold more sensitive than the conventional PCR assay. In addition, the LAMP reaction was carried out in a constant temperature environment, and temperature cycling is not required. So even using the temperature-controlled water bath could provide a heat stabile condition, while the higher precision of the PCR instruments is necessary for the PCR. Furthermore, using real-time turbidimetry provides a real-time display of the reaction condition, assuring less chance of contamination. Moreover, LAMP assay primers can specifically recognize a target sequence by 4 or 6 of the 6 or 8 independent target sequence regions, compared to PCR primers that recognize target sequences by two independent regions. Therefore, the LAMP assay is the most suitable technique for rapid detection of ALVs in clinical samples, especially for preliminary testing to determine whether ALVs exist.

A total of 81 tissue samples were collected from chickens suspected of ALV infection, which had died from hemangioma or dramatically weight loss. These samples were detected by the LAMP assay and by PCR and virus isolation methods. The positive rates were 77.8 % (63/81), 65.4 % (53/81), and 46.1 % (37/81) as determined respectively by the LAMP assay, conventional PCR, and virus isolation. Forty-four samples, previously described as being ALV-negative by means of using virus isolation were further analyzed by the LAMP assay and PCR. The results showed that 26 and 19 ALV-positive samples were detected, respectively. It is suggested that the sensitivity of the LAMP assay was the highest among the three methods. In fact, when the cells are cultured to the second generation, four cases of the cell culture supernatants, which judged negative by virus isolation but judged positive by both LAMP and PCR, showed positive results detected by ELISA again (data not shown). This is a normal phenomenon in regular virus isolate, because one limitation is that only samples containing live virus can be detected and sometimes samples containing just a minute amount virus need continuously passaging to grow to enough amount for detection. However, conventional PCR and the LAMP assay can detect both live viruses and replication-deficient viruses. Seven more samples were positively detected by the LAMP assay than the conventional PCR. It is suggested that the sensitivity of the LAMP assay was better than that of the PCR method in this application. In comparison to PCR as the current reference method, the SEN was 100 %, SPC was 73.7 %, PPV was 84.1 % and NPV was 100 % for the LAMP assay. For the ELISA assay, the SEN was only 76.8 %, NPV was 63.6 %. This indicated that false negative judgments cannot be avoided when using the ELISA assay but the LAMP assay can detect ALV at high sensitivity. In a sense, none of the assays is entirely acuracte. The eradication program for AL however needs assays with a higher sensitivity. The LAMP assay presented here meets this requirement and could help to avoid the spread of ALV caused by false-negative judgments.

A possible disadvantage of the LAMP assay is that it can be contaminated [29]. The LAMP reaction generates large amounts of pyrophosphate ion, and a white precipitate of magnesium pyrophosphate in the reaction mixture can be observed by the presence of turbidity monitored by real-time turbidimetry at 400 nm. Using real-time turbidimetry, the probability of false positives decreased compared with conventional visual inspection during the LAMP assay. On the one hand, our real-time turbidimetry assay could prevent the volatilization of the LAMP reaction solution because of heating. On the other hand, it can also avoid aerosol contamination due to not having to open the tube after the reaction.

The LAMP reaction generates large amounts of pyrophosphate ion, and a white precipitate of magnesium pyrophosphate in the reaction mixture can be monitored by real-time turbidimetry. When the turbidity of the sample exceeds the threshold it will judged as positive by real-time turbidimetry. Specificity and sensitivity results by visual inspection equaled the turbidity measurements, but we still recommend strongly using real-time turbidimetry to avoid aerosol contamination through opening of the tubes to add the dye.

The described method requires keeping the reagents strictly separated between the preparation room and the test room. Also, it is helpful not to open the lid as far as possible when the temperature-controlled water bath is used. In addition, a calcein dye or other dye like SYBR green dye was added after the reaction terminated (the reaction will be interfered with if the dye is added before the reaction starts) to better observe the results. This approach works well to avoid contamination. We also recommend precautions, such as a very clean environment for the preparation of the reaction mixture, and careful manipulation to avoid cross-contamination.

## Conclusions

In conclusion, we describe a LAMP assay for the detection of the common ALVs in chickens which is simple, rapid, sensitive, specific and inexpensive.

## Methods

### Ethics statement

None of the experiments in our study involved human participants.

### Virus

The DNAs of ALV-A (RAV-1), ALV-B (RAV-2), ALV-E (RAV-0), ALV-J (HPRS-103), avian reticuloendotheliosisvirus (REV), infectious laryngotracheitis virus (ILTV) and Marek’s disease virus (MDV) were obtained from the Harbin Veterinary Research Institute of Chinese Academy of Agricultural Sciences, which identified and ensured that they contained no other virus by classical PCR methods. Then, the viruses were stored at the Institute for Poultry Science and Health, Guangxi University.

### Clinical samples and treatment

A total of 81 clinical samples including livers and spleens of suspected ALV infected birds, including those showing hemangioma on the skin, were collected from each sampling sites (commercial chicken farms in Nanning, Yulin, Qinzhou, Baise and Liuzhou in Guangxi, China). Tissue samples were homogenized in phosphate buffered saline (PBS) containing a mix of the antibiotics penicillin and streptomycin, then centrifuged at 4 °C for 5 min at 6,000 × g. An aliquot of the supernatant was used to extract proviral DNA which was utilized as a template for the LAMP assay and routine PCR detection.

The samples’ supernatant was filtrated through 0.22 μm filters and inoculated into DF-1 cell cultures, which were plated with DMEM (GIBCO, NY) containing 10 % fetal bovine serum. Then incubation for 2 h, the cells were overlaid with 1 % fetal bovine serum and cultured in a 5 % CO_2_ atmosphere at 37 °C for 7 days. The cell culture supernatants were collected and used to detect *p*27 antigen through a commercial ELISA kit (IDEXX USA Inc., Beijing, China).

### DNA extraction

DNA was extracted from the clinical tissue samples using a UniversalGen DNA kit (CWBIO, Beijing, China), according to the manufacturer’s operation manual. These DNA extractions were stored at -20 °C utilized as a template for the LAMP assay and routine PCR detection.

### Conventional PCR

PCR assay for ALV-J detection was carried out with the subgroup-specific primers H5:H7(ALV-J) described by Smith et al [30]. The PCR primer sets for subgroups A, B, E are designated as H5: Cap A (ALV-A), H5: Cap B (ALV-B), H5: AD1 (ALV-E) , described by Zavala et al [31]. All PCR reactions were performed in accordance to the methods described for detection of ALV [31]. The PCR products were visualized in a 1.5 % agarose gel with ethidium bromide.

### LAMP primer designs

**Fig. 6:**
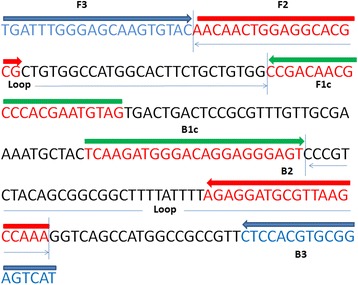
LAMP primers. The primers are indicated by arrows. Two inner primers (FIP and BIP) and outer primers (F3 and B3) are used in the LAMP method. FIP (BIP) is a hybrid primer consisting of the F1c (B1c) sequence and the F2 (B2) sequence. As represents a complementary sequence, the F1c (B1c) sequence is complementary to the F1 (B1) sequence. The regions which forms key loop structure in LAMP assay are also indicated

LAMP assay primers were designed using Primer Explorer V4 software (Eiken Chemical Co., Ltd., Tokyo, Japan) to specific amplified *pol* gene fragments of ALV-A, ALV-B and ALV-J, based on the region of the *pol* gene, which was conserved across ALV-A, ALV-B, ALV-E, and ALV-J and which would effectively avoid amplification of the EAV-HP family [30]. Before designing the specific primers, we compared the sequence identity of different ALV strains available in the GenBank and found conserved regions with more than 98 % accuracy. We then used BLAST analysis to check the specificity of the primers. Results showed that the primer bases were a good match for the ALV *pol* sequence, but not for other viruses. Then, the primers were synthesized by Guangzhou Invitrogen Co., Ltd. Outer primers were named F3 and B3, inner primers were named FIP and BIP (Table 3). The schematic diagram shows the location of each primer (Fig. 6); LAMP primers are indicated by arrows.

**Table 3:** LAMP primers sequence

Primer	Sequence (5’-3’)	Genome position
F3	TGATTTGGGGGCAAGTGTAC	4148-4167
B3	ATGACTCCGCACGTGGAG	4356-4373
FIP = F2+ F1c	CTACATTAGTGGGCGCTGTCGG-AACAACTGGAAGCACGCG	F2, 4168-4185 F1c, 4212-4233
BIP = B2 + B1c	TCAAGATGGGACAGGAGGGAGT-TTTGGCTTAACGCATCCTCT	B2, 4316-4335 B1c, 4267-4288

### LAMP assay

The LAMP reactions were carried out in 25 μl reaction mixture (DNA Amplification Kit; EIKEN CHEMICAL CO., LTD, Tochigi, Japan) containing the following reagents (final concentration):20 mM Tris-HCl (pH = 8.8), 10 mM KCl , 10 mM (NH4)_2_SO4, 0.1 % Tween20, 0.8 M betaine, 8 mM MgSO4, 1.4 mM dNTP each and 8U *Bst* DNA polymerase. The amount of primer needed for one reaction was 40 pmol for FIP and BIP, and 5 pmol for F3 and B3. Finally, an appropriate amount of template genomic DNA was added to the reaction tube. The reaction was carried out and monitored at 63 °C in a Loopamp real-time turbidimeter(LA320-C, Eiken Chemical Co., Ltd., Tokyo, Japan).
